# A workflow-driven approach to integrate generic software modules in a Trusted Third Party

**DOI:** 10.1186/s12967-015-0545-6

**Published:** 2015-06-04

**Authors:** Martin Bialke, Peter Penndorf, Tim Wegner, Thomas Bahls, Christoph Havemann, Jens Piegsa, Wolfgang Hoffmann

**Affiliations:** Institute for Community Medicine, Section Epidemiology of Health Care and Community Health, University Medicine Greifswald, Ellernholzstr. 1-2, 17487 Greifswald, Germany; Institute of Applied Microelectronics and Computer Engineering, University of Rostock, Rostock, Germany; German Centre for Cardiovascular Research (DZHK), Greifswald, Germany

**Keywords:** Medical data management, Data protection, Informed consent, Pseudonyms, Record linkage

## Abstract

**Background:**

Cohort studies and registries rely on massive amounts of personal medical data. Therefore, data protection and information security as well as ethical aspects gain in importance and need to be considered as early as possible during the establishment of a study. Resulting legal and ethical obligations require a precise implementation of appropriate technical and organisational measures for a Trusted Third Party.

**Methods:**

This paper defines and organises a consistent workflow-management to realize a Trusted Third Party. In particular, it focusses the technical implementation of a Trusted Third Party Dispatcher to provide basic functionalities (including identity management, pseudonym administration and informed consent management) and measures required to meet study specific conditions of cohort studies and registries. Thereby several independent open source software modules developed and provided by the MOSAIC project are used. This technical concept offers the necessary flexibility and extensibility to address legal and ethical requirements of individual scenarios.

**Results:**

The developed concept for a Trusted Third Party Dispatcher allows mapping single process steps as well as individual requirements and characteristics of particular studies to workflows, which in turn can be combined to model complex Trusted Third Party processes. The uniformity of this approach permits unrestricted re-combination of the available functionalities (depending on the applied software modules) for various research projects.

**Conclusion:**

The proposed approach for the technical implementation of an independent Trusted Third Party reduces the effort for scenario specific implementations as well as for maintenance. The applicability and the efficacy of the concept for a workflow-driven Trusted Third Party could be confirmed during the establishment of several nationwide studies (e.g. German Centre for Cardiovascular Research and the National Cohort).

## Background

Epidemiological research in the context of cohort studies and registries becomes increasingly cooperative and often requires multi-site acquisition of extensive medical data. As a consequence research becomes more and more networked regarding communication, information exchange and cross-coordination between participating research institutions, laboratories and imaging facilities.

For these reasons, legal aspects of data security and information protection significantly gain in importance. This concerns the written informed consent of potential participants, which is mandatory for acquiring medical data for research purposes from an ethical point of view. On the national level legal principles like data avoidance and frugality [§3a of the German Federal Data Protection Act (Bundesdatenschutzgesetz, BDSG)] as well as requirements for the separation of identifying data from further personal data (§40 BDSG) need to be accounted for. International legislation includes the “Convention for the Protection of Individuals with regard to Automatic Processing of Personal Data” (Council of Europe) [1], the “EU legal framework on the protection of personal data” (European Commission) [2] and the “Declaration of Helsinki” (World Medical Association) [3]. The resulting legal and ethical obligations require effective solutions realizing all necessary measures for data protection and IT security.

In Germany the *Technology, Methods and Infrastructure for Networked Medical Research* (TMF) provided a guideline [4] proposing a Trusted Third Party (TTP) to address typical challenges in data protection and ethics. Following the TMF-specification a TTP requires an informational separation of powers by separating person identifying information (PII) and medical information from a technical as well as from an organizational perspective. This includes an electronic identity-management and should be supplemented by a secure pseudonymisation mechanism [4]. Following this definition, a TTP is described as a combination of technical as well as organisational measures and shall comply with fundamental principles according to data protection rules for IT-solutions [4]. Moreover, the guideline demands the TTP to be legally, staff-wise and spatially autonomous and independent.

It is of importance that the employees of a TTP (the data trustee) do not depend on the institutions which are providing or processing the research data. In particular the employees need to be independent in terms of their contracts, incomes, duties, work hours and other operational aspects from all scientists of the project that they support. This can be realized either in a separate legal organisation or on a contract level. According to TMF guidelines [4] and legal reports [5] as well as the Federal Data Protection Act (cf. §28 BDSG) the processing of data on a contract level prevents a sufficient informational separation of powers. From an organisational perspective the TTP requires a functional transfer (transfer of full responsibilities for data processing) in order to be independent of instructions from the initiators of a research.

**Figure 1:**
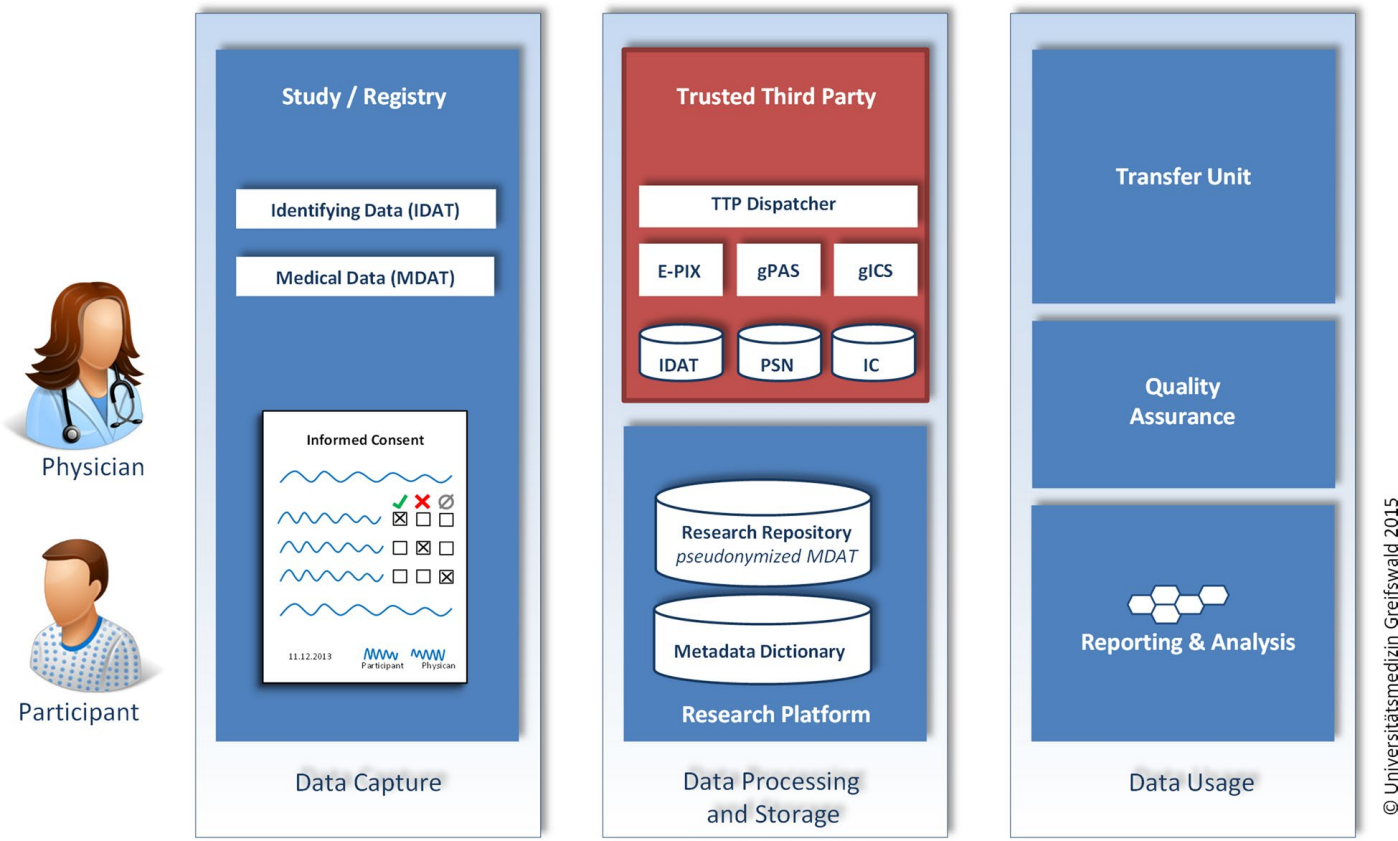
The Trusted Third Party (TTP) is a core element in a research data management infrastructure: After provision of a system-wide unique identifier for the study participant (identity management), the TTP stores the Informed Consent Document (IC) and provides the required pseudonyms (PSN) for data capture. The participants identifying data (IDAT) is stored within the TTP. Pseudonymised medical data (MDAT) and related metadata are stored in the research platform. The usage of (pseudonymised) medical data typically includes quality assurance, reporting and analysis. If the participant consented to the secondary use of his medical data, data may be provided for further research projects via a separate transfer unit.

Along these guidelines an independent TTP was established at the Institute for Community Medicine at the University Medicine Greifswald to exclusively handle participant identifying data, which have been separated from all further informative variables including metadata. The TTP centrally provides the necessary data protection functionalities and measures for various studies and registries. Figure 1 presents an overview of a typical TTP infrastructure and involved stakeholders in the context of research data management.

This paper focusses the technical implementation of a TTP. The goal is to define and organise a consistent workflow-management within the TTP. Allowing to increase reusability for individual TTP scenarios, the workflow approach shall reduce the effort for implementation and maintenance.

## Methods

### Assembling a modular Trusted Third Party

The spectrum of tasks of a data trustee includes the management of identities, informed consents and the generation of pseudonyms. Additionally, the data trustee supports the matching of personal data from population registries and further external data sources.

An *identity management* is required to manage participants and assigned participant identities. It includes probabilistic matching algorithms for an efficient and fault-tolerant record-linkage. Furthermore, it comprehends the provision and management of appropriate pseudonyms for each set of identities. Especially in prospective cohort studies and registries compiling variations in the identifying data of a participant (a so called identity), e.g. different spelling in a participant’s name, need to be stored.

To ensure compliance to the principles of informational self-determination [4], the participant has to be able to consent to several aspects of data processing. Within the TTP the *management of informed consents* includes the provision of patient information documents, the consent itself and a monitoring of various types of revocations. For digital processing informed consent documents are depicted as modular examinable policies and modules and are combined with additional data like electronic signatures, dates and organisational information. This modular informed consent allows for verifiable as well as contemporary statements, whether for example the processing of a participant’s data, the secondary use of collected data or the specimen-collection is legitimate or not.

The efficient generation and *administration of pseudonyms* within the TTP is a key functionality when medical scientific data needs to be processed and permanently stored. In order to provide scientific data for research projects and secondary use, the data has to be pseudonymised secondarily or be anonymised. In some cases an anonymisation is not applicable. Follow-up investigations, the communication of incidental findings or the linkage of secondary data require the pseudonymisation to be reversible in order to retrieve the corresponding participants for further contact.

For the implementation of the independent TTP several open source software modules are used. Following the basic concepts and processes described by the TMF [4], the MOSAIC project [6] (funded by the German Research Foundation (*HO 1937/2*-*1*)) has developed a set of practical tools to address data protection challenges and to provide support for the implementation of a data management in epidemiologic research projects. These free software tools (E-PIX, gICS, gPAS) facilitate the principles of “privacy by design” [7] and use uniform technical standards. Moreover these tools provide a service-oriented architecture and consistent graphical user interfaces.

The *E-PIX (Enterprise Patient Identifier Cross Referencing)* [8] allows a precise identity management and supports the data trustee to distinguish participants sustainably based on their identifying data (IDAT). It follows the principles of a Master Person Index. This ensures a participant to exist only once in the linkage database based on demographic information [9]. The completely service-based software module generates a unique identifier for every managed participant and allows solving ambiguous matching cases interactively using a web-based graphical interface. The equally modular solution *gPAS (generic Pseudonym Administration Service)* [10] adopts similar technical approaches and provides domain-specific pseudonym creation, de-pseudonymisation and anonymisation functionalities. The utilisation of *gICS (generic Informed Consent Service)* [11] completes the set of TTP tools. It facilitates the management of digital informed consent documents and allows automatable checks for consent validity and revocations [11]. Modular informed consents are defined, based on examinable policies and re-usable modules.

The simultaneous use of the MOSAIC software modules E-PIX, gICS and gPAS allows implementing basic requirements of an independent TTP. The administration of participant identities, informed consents and pseudonyms can be performed using graphical web interfaces. However, due to their modular design there is no direct communication among these components. In order to realize more complex workflows, a manual intervention of the data trustee is necessary in many tasks. For example, if a new participant is recruited, it is necessary to assign a unique identifier based on his IDAT (identity management), to pseudonymise this unique identifier (pseudonym administration) and to return the generated pseudonym in order to start capturing the medical data within the study site.

Most widely automating the communication between the software-modules E-PIX, gPAS and gICS through well-defined workflows reduces the number of necessary manual interventions of the data trustee. Only a small number of crucial decisions remains, where a human interaction cannot be replaced (e.g. to evaluate and resolve possible matches).

### Extending flexibility to support individual scenarios

The required communication between the previously described TTP services depends on the workflow of a specific cohort or registry and, hence, individual characteristics may differ from the typical scenario. In order to flexibly orchestrate the particular TTP services and to coordinate the corresponding communication between the services, a dispatcher has been developed. The *TTP Dispatcher* represents the conceptual continuation of a request dispatcher, which was introduced in the GANI_MED project. [12].

**Figure 2:**
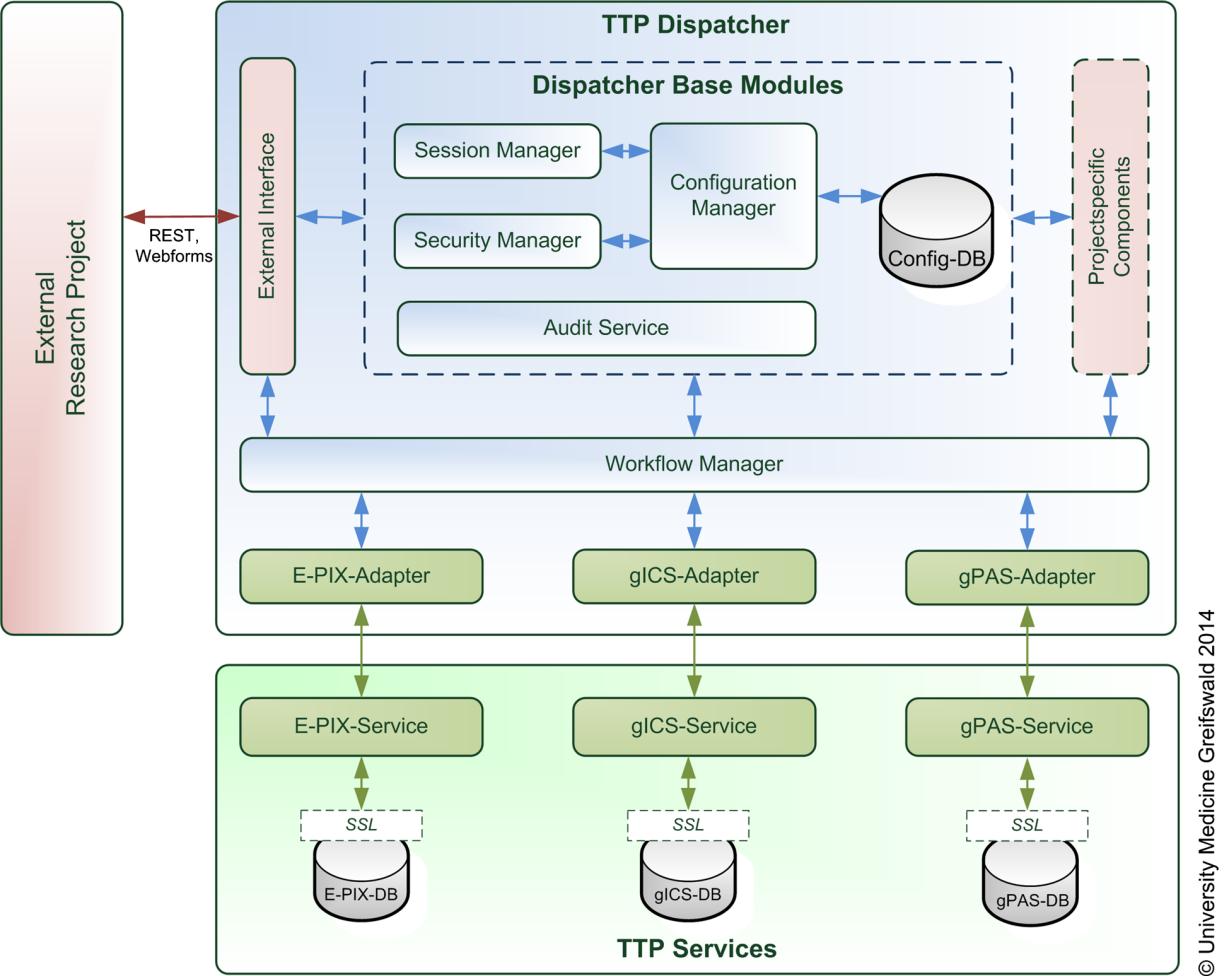
Component Overview of the Trusted Third Party (TPP) Dispatcher: Composing functionalities from independent service modules (*E-PIX* Identity Management, *gICS* Informed Consent Management, *gPAS* Pseudonym Management).

Figure 2 presents an overview of the implemented components and defines the specific data flows. The TTP Dispatcher consists of several modules and communicates with the previously described *TTP service* modules gPAS, gICS and E-PIX via corresponding *Service Adapters*. The service adapter connects the TTP Dispatcher with the respective module. This approach enhances the interoperability of the TTP implementation and reduces the technical effort to add further software modules if necessary. Moreover, using service adapters facilitates and standardises the dispatcher-internal handling of the utilized service functionalities. The *Configuration Manager* administrates project-specific and dispatcher-specific configurations in a separate database. This includes workflows, roles and rights as well as a set of individual parameters (e.g. for informed consents or data entry validation). The *External Interface* allows connections to selected functionalities for external use in order to integrate the TTP Dispatcher in external applications. It can be accessed using the representational state transfer protocol (REST) or web-forms. The external interface and concept is oriented towards an existing identity management solution (the “Mainzelliste” [13]). The specification was extended in cooperation with the authors. Another key component is the *Session Manager*, which handles external requests and administrates all required information. In order to grant access to available functionalities for registered users and systems only, the *Security Manager* provides a basic role-and-rights-management. The TPP Dispatcher is not limited to the components in Figure 2. *Project specific components*, e.g. to process data from smart cards, sign pads for electronic signatures, individual web forms as well as additional databases, can be readily integrated. The administration of project specific workflows is handled by the *Workflow Manager*.

### Flexibility through workflows

In terms of a TTP, a workflow technically describes a sequence of (parallel) processes and operations, starting with an input and ending with a defined outcome. Workflows are being used to control and process the necessary calls to the connected software modules E-PIX, gPAS and gICS. They are distinguished into groups. Basic workflows represent common tasks of a data trustee and are of relevance in most project scenarios, e.g. checking if a participant already exists in the management system or generating pseudonyms for a list of participant identifiers. Project-specific workflows describe all necessary individual processes and operations beyond, for example all required steps to automatically generate a pseudonym when a new participant is created in a study site based on his IDAT and a valid informed consent. The separation of basic and project-specific workflows allows a consistent approach for several implementations of the TTP Dispatcher. This architecture hereby supports portability to other research projects, reduces maintenances and improves the sustainability of a TTP implementation.

The technical description of each workflow is performed using Apache Camel [14]. Based on Enterprise Integration Patterns [15] routes can be defined using a domain specific language. Each route comes with at least two end-points (source and target, e.g. a simple file, a web-service or an internal process), which are expecting an input (e.g. objects, messages) and returning a result. These end-points are linked using a message channel and basic elements of the Apache Camel syntax.

The starting point of a workflow defines the origin of an object or message (“from”). Combined with several processing steps (manual input, conversion), simple criteria-based conditions and a target point (“to”) a workflow is specified. The necessary information (e.g. unique identifiers, pseudonyms or informed consent data) is passed directly between the individual workflow steps. The TTP Dispatcher comes with a basic set of predefined workflows (see Table 1 for details), which can also provide a basis for additional project-specific workflows.

**Table 1:** Overview of basic Trusted Third Party workflows

Workflow	Description
get_mpi	Generate MPI ID for given IDAT using E-PIX-service
check_patient_exists	Check if a participant with given IDAT already exists in the E-PIX-database
get_id_from_id	Get a pseudonym for a given identifier (e.g. MPI ID) and vice versa using the gPAS-service
add_consent	Add a new informed consent (based on a template containing several modules and policies) for the given identifier using the gICS-service
check_consent_exists	Check if an informed consent for the given identifier exists in the gICS-database
query_consent	Query a list of policies and their consented state for a given informed consent identifier using the gICS-service
add_scan	Add a document scan to a previously defined informed consent using the gICS-service
update_participant	Update a participants IDAT already existing in the E-PIX-database
get_participant_by_mpi_id	Retrieve a participants IDAT from the E-PIX database identified by its MPI ID
add_participant_get_psn	Sequential workflow combining get_mpi and get_id_from_id
get_participant_by_psn	Sequential workflow combining get_id_from_id and get_participant_by_mpi_id

## Results

An essential part for the technical establishment of a Trusted Third Party is the implementation of required dispatcher functionalities. In the past the necessary individual implementations for a cohort study or registry required up to 6 month of work.

**Figure 3:**
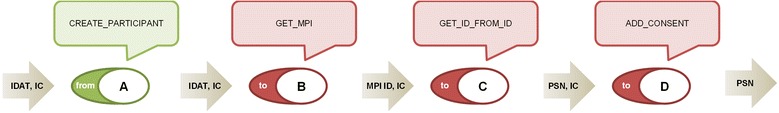
Example: defining the individual workflow “Create Participant” for the DZHK required only a subset of basic workflows.

Using the proposed workflow-driven approach allows to accomplish the necessary customisations within weeks, by reusing various basic workflows and combining them as intended. For example creating a new participant (cf. Figure 3) in a study site of the DZHK, based on his IDAT and a valid informed consent (A), requires the combination of three basic workflows for full process automation: For record linkage the given IDAT of the participant are passed to the corresponding basic workflow using the E-PIX service (B). The result is a unique identifier which will be pseudonymised using the gPAS-Service subsequently (C). Finally the pseudonymised informed consent document is stored using the gICS-Service (D) and the TTP Dispatcher returns the pseudonym to the study site. Within the study site, the capture of medical data can begin.

In case of an error, the workflow processing is interrupted. Among other information, the error message and the error origin are documented and returned to the respective study site.

As the example demonstrates, a consistent workflow management allows easily linking available functionalities (E-PIX, gICS, gPAS) by reusing and combining predefined workflows. Thus the individual character of cohort studies and registries can be depicted straightforward. Moreover, study specific processes are most widely automatable and manual intervention of the data trustee could be essentially reduced.

## Discussion

The components and workflows of a TTP vary according to their specific context. For example, the Central Clinical Cancer Registry in Mecklenburg-Western Pomerania [16] does not require a consent management and the German National Cohort [17] uses a specific pseudonymisation for different sites and data categories (e.g. MRT, bio samples, web-forms).

Implementing a TTP on a basis of tools without uniform interfaces and with varying technical standards, demands an advanced IT knowledge and requires massive implementation efforts. That is why using the MOSAIC modules [8, 10, 11] seems to be a suitable and practical approach. Furthermore, using a workflow-based dispatcher in order to coordinate the specific functionalities [12] allows a consistent tailoring and adoption to new projects. Table 2 lists resulting advantages and disadvantages of the developed TTP Dispatcher.

**Table 2:** Advantages and disadvantages of the developed Trusted Third Party Dispatcher

Advantages	Disadvantages
Support for automation reduces susceptibility to errors and accelerates internal TTP processes	Initial configuration of the TTP Dispatcher requires professional IT support to set up mandatory databases and the application server
Integrated audit-and-trail-mechanisms ensure traceability and transparency of participating systems	Changes and updates in TTP Dispatcher core functions involve a determined update management including tests in the respective project, study or registry
Modular and adaptable workflows improve portability and re-usability	
Multi-client capability to manage large multi-site projects	
Interoperability of service components	

Following the described approach for the technical implementation of a Trusted Third Party supports compliance with project-specific legal data protection requirements in cohort studies and registries. But in order to exhaustively fulfil security, data protection, ethical and legal requirements [4], additional measures are necessary. Among others, this includes the institution of a data trustee, several dedicated rules, access controls for non-employees, separated network infrastructures and full client-capability on a technical and organisational level [18], resulting in the separated storage of participant identifying data for each supported study and registry. Moreover, regular internal and external audits have to be engaged involving both the institutional and the federal data protection officers.

The aim of the described TTP Dispatcher approach has a significant difference to existing IT platforms supporting clinical research, such as EHR4CR [19]. The proposed TTP approach focusses exclusively on the management of participant identifying data and related technical and organisational measures. Medical data is not processed within the TTP. EHR4CR focusses a widespread support to all steps of a clinical trial process instead. This includes the provision of information about new and running trials, several tools for data managers and investigators, the provision of query engines, recruitment software, an identity and access management, as well as a security framework. Unlike the proposed TTP approach, the EHR4CR IT platform stores aggregated medical information and patient identifying data does not leave the clinical context.

The concept of the TTP Dispatcher has already seen successful implementation in the German Centre for Cardiovascular Research (DZHK) [20] and the German National Cohort [17]. The resulting pattern can flexibly be adopted and easily be extended for reuse in future cohort studies and registries. The established TTP solutions are compatible to legal requirements of the “Convention for the Protection of Individuals with regard to Automatic Processing of Personal Data” (Council of Europe) [1], the “Declaration of Helsinki” (World Medical Association) [3], the “EU legal framework on the protection of personal data” (European Commission) [2] as well as the “Treaty of Lisbon” (European Union) [21] and they are aligned to the previously mentioned recommendations of the TMF data protection concepts for medical research [4].

## Conclusions

During the recruitment of participants for cohort studies and registries particularly the acquisition, processing and storage of personal health data necessitate both compliance with ethical standards and stringent policies for data protection. For Germany, the resulting requirements for data management are compiled comprehensively in the guideline provided by the TMF [4]. Conformity is usually achieved by the implementation of a Trusted Third Party (TTP). However, the individual TTP implementation for different studies is associated with considerable high technical efforts that can be prohibitive in smaller studies or in institutions without a professional IT-department.

This paper demonstrates how generic software modules developed and provided by the MOSAIC project [8, 10, 11] can be deployed in order to meet essential TTP requirements. The concept of a workflow-driven dispatcher is introduced combining these modules in structured workflows, allowing for a free combination of separate functionalities. Single process steps can be easily implemented by concatenating corresponding function calls and mapping them to workflows. The combination of multiple workflows enables an efficient conception and implementation of highly complex working procedures. Simultaneously the necessary effort for customisation is reduced to a minimum.

The proposed approach for the technical implementation of a TTP facilitates the necessary flexibility, portability and reusability for application in cohort studies and registries. This is achieved by mapping the individual requirements and characteristics of a particular study to pre-defined workflows. Reusability additionally benefits from the encapsulation of module logic and a uniform interface for all modules avoiding study specific modifications of individual modules or functionalities. The generic software modules connected with the workflow approach presented in this paper can easily be adopted to accommodate national and international requirements in terms of informed consent, identity management, pseudonymisation, data linkage and data transfer.

However, specification of a uniform interface for essential functionalities and parameters accounting for established standards and methods within the scientific community must still be considered time-consuming and labour-intensive. Future work will focus on further facilitating the establishment of an independent TTP, including workflow visualization, a generic module configuration independent from the deployed services, a graphical configuration tool for the configuration of the dispatcher and an extended central role-and-rights-management.

## Abbreviations

DZHK: Deutsches Zentrum für Herz-Kreislauf-Forschung (German Centre for Cardiovascular Research)
E-PIX: Enterprise Patient Identifier Cross-referencing
gICS: generic Informed Consent Administration Service
gPAS: generic Pseudonym Administration Service
IDAT: identifying data
MDAT: medical data
PSN: pseudonym
MPI ID: unique identifier following concepts of a Master Patient Index
TMF: Technology, Methods and Infrastructure for Networked Medical Research
TTP: Trusted Third Party
